# Time-Resolved Measurement of the ATP-Dependent Motion of the Group II Chaperonin by Diffracted Electron Tracking

**DOI:** 10.3390/ijms19040950

**Published:** 2018-03-22

**Authors:** Naoki Ogawa, Yohei Y. Yamamoto, Keisuke Abe, Hiroshi Sekiguchi, Yuji C. Sasaki, Akira Ishikawa, Judith Frydman, Masafumi Yohda

**Affiliations:** 1 Department of Biotechnology and Life Science, Tokyo University of Agriculture and Technology, Naka, Koganei, Tokyo 184-8588, Japan; nogawa@phys.chs.nihon-u.ac.jp (N.O.); yohei.yamamoto@yohda.net (Y.Y.Y.); keisuke.abe@yohda.net (K.A.); 2 The Institute of Natural Sciences, College of Humanities and Sciences, Nihon University, Sakurajosui, Setagaya, Tokyo 156-8550, Japan; ishiaki@mti.biglobe.ne.jp; 3 Research & Utilization Division, Japan Synchrotron Radiation Research Institute, Sayo, Hyogo 679-5198, Japan; sekiguchi@spring8.or.jp; 4 Graduate School of Frontier Sciences, University of Tokyo, Kashiwanoha, Kashiwa, Chiba 277-8561, Japan; ycsasaki@k.u-tokyo.ac.jp; 5 Department of Biology, Stanford University, Palo Alto, CA 94305, USA; jfrydman@stanford.edu; 6 Institute of Global Innovation Research, Tokyo University of Agriculture and Technology, Naka, Koganei, Tokyo 184-8588, Japan

**Keywords:** chaperone, chaperonin, folding, single molecule, dynamics

## Abstract

Previously, we demonstrated the ATP-dependent dynamics of a group II chaperonin at the single-molecule level by diffracted X-ray tracking (DXT). The disadvantage of DXT is that it requires a strong X-ray source and also perfect gold nano-crystals. To resolve this problem, we developed diffracted electron tracking (DET). Electron beams have scattering cross-sections that are approximately 1000 times larger than those of X-rays. Thus, DET enables us to perform super-accurate measurements of the time-resolved 3D motion of proteins labeled with commercially available gold nanorods using a scanning electron microscope. In this study, we compared DXT and DET using the group II chaperonin from *Methanococcus maripaludis* (MmCpn) as a model protein. In DET, the samples are prepared in an environmental cell (EC). To reduce the electron beam-induced protein damage, we immobilized MmCpn on the bottom of the EC to expose gold nanorods close to the carbon thin film. The sample setup worked well, and the motions of gold nanorods were clearly traced. Compared with the results of DXT, the mobility in DET was significantly higher, which is probably due to the difference in the method for immobilization. In DET, MmCpn was immobilized on a film of triacetyl cellulose. Whereas proteins are directly attached on the surface of solid support in DXT. Therefore, MmCpn could move relatively freely in DET. DET will be a state-of-the-art technology for analyzing protein dynamics.

## 1. Introduction

Chaperonins are essential molecular chaperones with a characteristic double-ring structure composed of subunits with a molecular weight of approximately 60 kDa [1,2]. Chaperonin captures an unfolded protein and folds it in an ATP-dependent manner. Chaperonins are divided into two groups (I and II) based on their structure and amino acid sequences [3,4]. Group I chaperonins, which are known as Hsp60 or GroEL, exist in bacteria, mitochondria, and chloroplasts. They function with a co-chaperonin, Hsp10 or GroES, which constitutes the lid of the folding cage. The chaperonins of archaea or the eukaryotic cytosol belong to the group II chaperonins which have the built-in lids formed by helical protrusions in the apical domain [5,6,7].

Group II chaperonins capture an unfolded protein in the central cavity of a ring in the open state, and folding is mediated by the ATP-dependent closure of the lid [8,9]. Structural studies suggested that the ring structure of the chaperonin twists to seal off the central cavity [10]. We analyzed the ATP-dependent dynamics of the archaeal group II chaperonin at the single-molecule level by diffracted X-ray tracking (DXT) and revealed ATP dependent rotational motion [11]. DXT allows direct picometer-scale observation of individual biomolecules in both real time and real space [12,13]. The results have clearly shown that DXT is a powerful tool for analyzing protein motions. However, there are some disadvantages. The method requires a strong X-ray source, such as that at synchrotron radiation facilities. Therefore, for the tracking method to be more available to ordinary biochemical researchers, we have developed diffracted electron tracking (DET) [14,15]. In DET, the orientation of a nanocrystal linked to a single protein molecule is analyzed by monitoring an electron back scattering diffraction pattern (EBSP) using a wet-scanning electron microscope (SEM). Electron beams have scattering cross-sections that are 1000 times larger than those of X-rays. The use of X-rays in DXT necessitates the use of a perfect crystal. On the contrary, DET can use commercially available gold nanoparticles because the electron probe is only sensitive to their surface structure. The EBSP presents a line pattern, which is in contrast to the spot pattern of the diffraction phenomena of X-rays. This trait enables DET to observe the high-speed anisotropic motion of an individual gold nanoparticle in three-dimension at the picometer scale.

To examine the motion in aqueous solution, an environmental cell (EC) was used in wet-SEM. The ECs were covered with a 150 µm diameter single-hole grid for windows to allow the passage of electrons. These windows were sealed with a carbon film (20 nm in thickness). The carbon film acted as a barrier against atmospheric air pressure from the high-vacuum conditions of the SEM column. In our previous study, the gold nanoparticles were attached to the carbon film. We were able to collect time-resolved, 3D, super-accurate (picometer scale) motion tracking data on single nanoparticles under vacuum and in solution [14].

**Figure 1:**
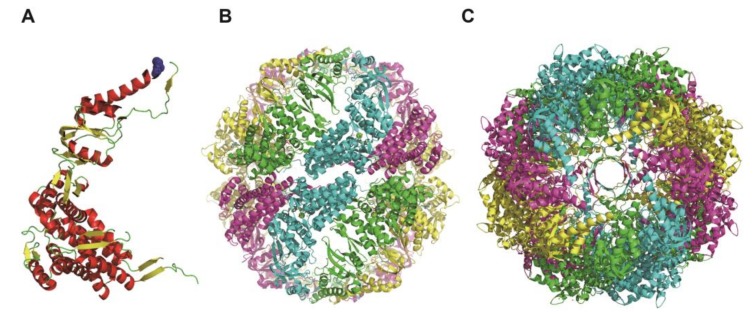
Structure of MmCpn. (**A**) Structure of MmCpn monomer. K255 residue was shown by spherical model. α-helices and beta-sheets are colored red and yellow, respectively. (**B**) The side view of MmCpn 16mer. (**C**) The top view of MmCpn 16mer. Each subunit is shown in the different color. The images are made from the crystal structure data (PDB ID: 3ruq) using PyMol (Schrödinger; Cambridge, MA, USA).

In this study, we examined the ATP-dependent motion of group II chaperonin by DET. In our previous studies, we used the group II chaperonin from a hyperthermophilic archaeon, *Thermococcus* sp. strain KS1 (TKS1-Cpn) because TKS1-Cpn exhibits high protein folding ability and ATP-dependent mobility [16,17]. The disadvantage of TKS1-Cpn is that it can function only at high temperatures (greater than 50 °C). We can perform DXT experiments at an elevated temperature because the cell for DXT is completely sealed. On the contrary, we must perform the DET experiment at room temperature because of the lack of a heating system in our system and the relative fragility of the thin carbon film of the EC. Therefore, we selected the group II chaperonin of the mesophilic archaeon *Methanococcus maripaludis* (MmCpn), which functions and exhibits conformational change at room temperatures (Figure 1) [18,19].

MmCpn is also the most-studied group II chaperonin. Its ATP-dependent conformational change has been studied in detail [10,20,21]. In this study, we compared the results of DXT and DET using MmCpn as a model protein. The results clearly showed the advantage of DET.

## 2. Results

**Figure 2:**
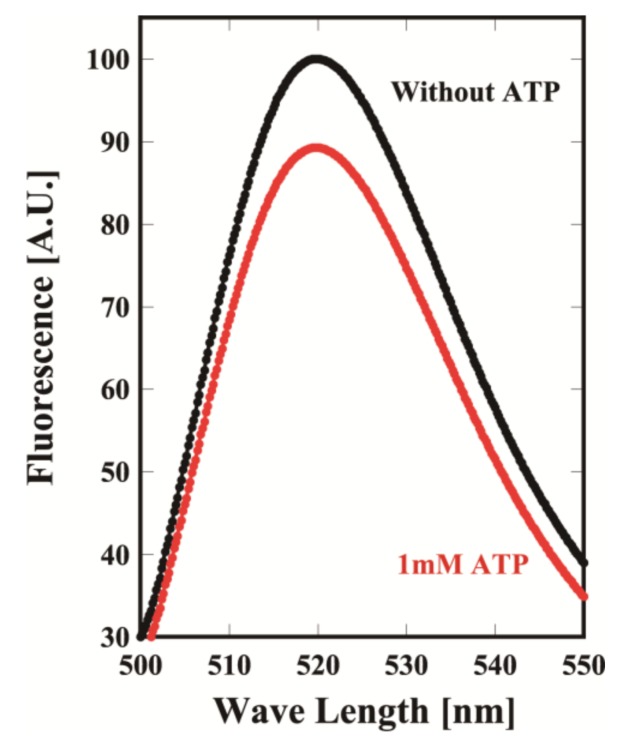
Fluorescence spectral change of the fluorescein-labeled MmCpn. MmCpnP255C was labeled with fluorescein, and the fluorescence spectra were measured using excitation at 493 nm at 29 °C (Black) without ATP with 1 mM ATP (Red). The details are described in the Materials and Methods. A.U.: arbitrary unit.

To perform DXT and DET experiments, a Cys residue was added to the tip of the helical protrusion for binding with a gold nanoparticle and immobilization. In addition, to avoid nonspecific binding with gold nanoparticles, other Cys residues were removed. For that purpose, Pro255 or Lys256 of the Cys-less mutant of MmCpn was changed to Cys (MmCpnK256C, MmCpnP255C). The final versions contain the amino acid replacements C140M, C237E, P255C, C286V, C359V, C393S, C470Y and C484T in addition to K256C or P255C. The mutants were expressed and purified using nearly the same procedure as for the wild type [21]. They were purified to homogeneity in SDS-PAGE. To examine whether the mutants could change the conformation in an ATP-dependent manner, MmCpnK256C was labeled with fluorescein at Cys256 and used in a fluorescence intensity assay with or without ATP. The fluorescence of fluorescein decreased when ATP was added (Figure 2). The results clearly showed that MmCpnK256C changed from the opened to closed conformation in an ATP-dependent manner. Thus, we used the mutants for DXT and DET analyses.

**Figure 3:**
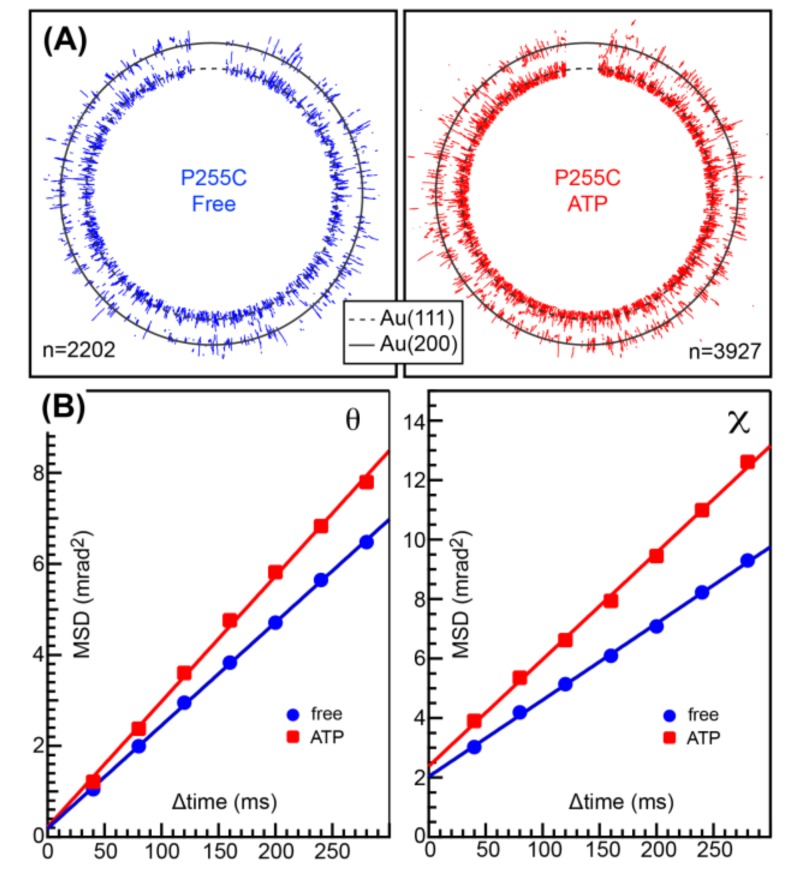
ATP-dependent rotational motion of MmCpnP255C tracked by DXT. (**A**) DXT traces of gold nanocrystals immobilized on the ring of MmCpnP255C in the absence (**left**) and presence of ATP (**right**). (**B**) Mean square angular displacement (MSD) of the *θ* (**left**) and *χ* (**right**) directions in the presence and absence of ATP as a function of the time interval (*Δ*time) in the presence of 0 mM ATP and 1 mM ATP at 30 °C.

MmCpnP255C or MmCpnK256C was immobilized on a gold-coated substrate surface by the gold-thiol bond. Then, a gold nanocrystal (nanoparticle) was attached to the other side of the double ring. Because MmCpnP255C showed better results compared to MmCpnK256C in the preliminary experiment, we used MmCpnP255C for further studies. The twisting and tilting of MmCpnP255C corresponded to Laue spots from the gold nanocrystals in the concentric circle (*χ*) and radial (*θ*) directions, respectively. Figure 3A shows traces of Laue spots from gold nanocrystals on MmCpnP255C in the absence (left) or presence of 2 mM ATP (right). Compared with previous observation on TKS1-Cpn, the concentric circle motion was not clearly observed. Then, we compared the mean square angular displacement (MSD) in the *θ* and *χ* directions as a function of the time interval (*Δ*time) in the presence and absence of ATP (Figure 3B). MSDs represent the deviations of the value with respect to a reference value over time. The MSD curve showed the slight activation of MmCpnP255C motion both in tilting (*θ*) and twisting (*χ*). The differences are also shown by the angular/rotational diffusion constants (Table 1). However, compared with our previous results for TKS1-Cpn, the activation was modest [11].

**Figure 4:**
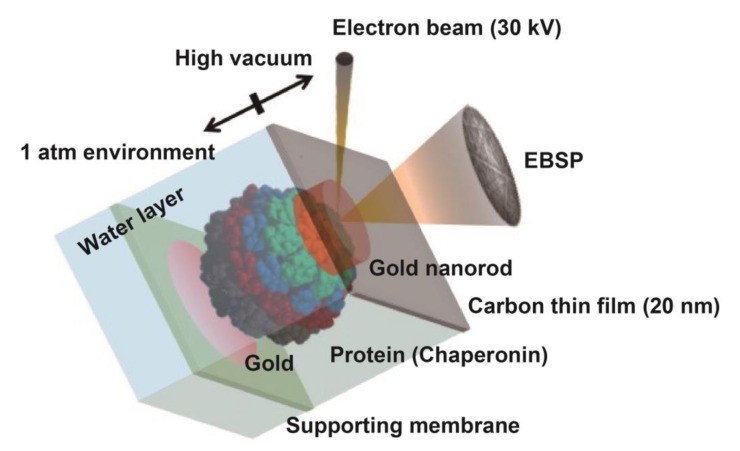
Schematic image of the experimental setup for diffracted electron tracking (DET). MmCpnP255C was immobilized on the supporting membrane in the EC, and a gold nanorod was attached to the other side of chaperonin. The EC was sealed by a carbon thin film. An electron beam irradiated through the carbon thin film, and the EBSP was detected.

Then, we tried to analyze the motion by DET. In our previous experiment, gold nanoparticles were immobilized on a carbon sealing film via triacetyl cellulose (TAC) support. When we immobilized MmCpnP255C on the carbon sealing film, the gold nanoparticle was located on the other side of the carbon film. In such condition, the electron beam would irradiate the gold nanoparticle through MmCpnP255C, which would cause severe electron beam damage to MmCpnP255C. Therefore, we immobilized MmCpnP255C on the TAC support, and gold nanorods were attached to MmCpnP255C. Thus, a prepared TAC support was placed in the EC containing TKM buffer (50 mM Tris-HCl, pH 7.5, 100 mM KCl, and 25 mM MgCl_2_) with or without 1 mM ATP (Figure 4). Then, the EC was covered with a carbon thin film. The electron beam passed though the carbon thin film and buffer and then irradiated the gold nanorod. The effect of the electron beam on MmCpnP255C should be a slight one.

**Figure 5:**
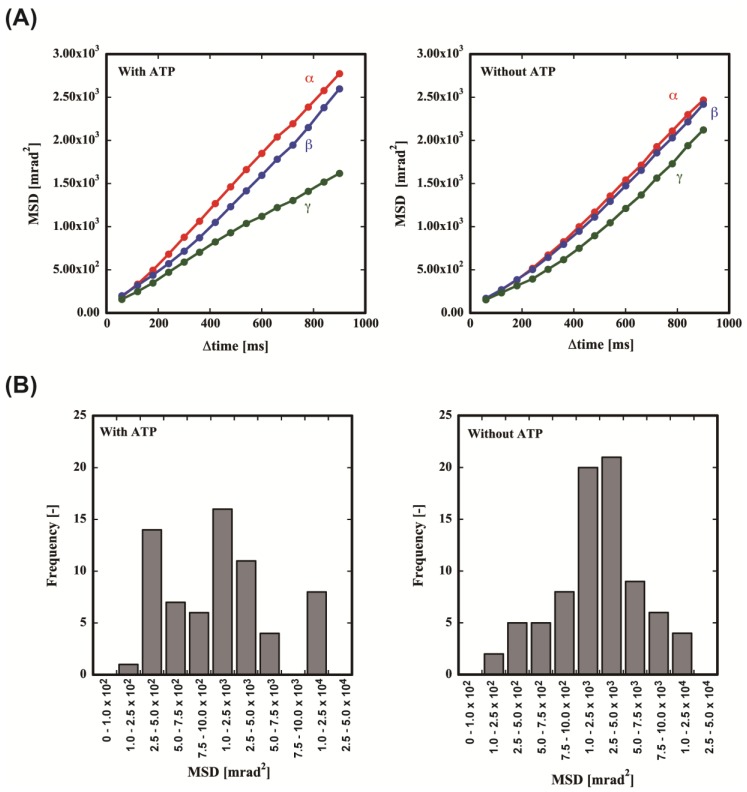
ATP-dependent rotational motion of MmCpnP255C tracked by DET. (**A**) MSD against the time interval. The MSD curves of the gold nanorods observed as a function of time interval (*Δ*time) with or without ATP. (**B**) Distribution of the absolute angular displacement of the rotation angle (**MSD**). The details are described in the Materials and methods.

From the EBSP dynamics, the orientation of the crystallographic axis of the gold nanorod was determined. For adjacent frames, we measured the rotation angles *α*, *β*, and *γ* of the principal lattice vectors ***a***, ***b***, and ***c***, respectively, of each gold nanorod. To analyze the motion of the gold nanorod, we plotted the amplitude mean-square displacement (MSD) curves of the values of *α*, *β*, and *γ* as a function of the time interval (*Δ*time) with or without ATP (Figure 5A). Without ATP, almost uniform rotational motion was observed in all axes. On the contrary, ATP induced significant asymmetry in the motion. The rotational motion for the *α* and *β* axes was significantly enhanced. However, the motion along the *γ* axis was almost the same as that without ATP. This result means that the motion without ATP is Brownian motion and ATP induces the rotational motion of MmCpnP255C.

The amplitude of the rotation angle (*ω*) is calculated from the orientation matrix ***g***, which is defined as follows.

(1) $$\left(\begin{array}{l} a\\ b\\ c \end{array}\right)=\left(\begin{array}{ccc} l_{a} & m_{a} & n_{a}\\ l_{b} & m_{b} & n_{b}\\ l_{c} & m_{c} & n_{c} \end{array}\right)=g\left(\begin{array}{ccc} 1 & 0 & 0\\ 0 & 1 & 0\\ 0 & 0 & 1 \end{array}\right)$$

The single-axis rotation between the *i*_th_ and *j*_th_ time step (*ω_ij_*) is calculated as follows.

(2) $$2cos\omega_{ij}=Trance{\left(g_{j}g_{i}{}^{-1}\right)}^{-1}$$

The MSD of *ω* indicated a slight increase in mobility in the presence of ATP. A significant difference was observed in the histograms (Figure 5B). Without ATP, the frequency seems to follow a Gaussian distribution. On the contrary, the frequencies for the high and low mobility were significantly increased in the presence of ATP. This result clearly demonstrates that the motion is not only random, but also includes ATP-dependent motion (Figure 5B).

## 3. Discussion

In this study, we first analyzed the motion of proteins by DET. Our previous studies have shown that group II chaperonins exhibit the clearest results in DXT experiments. We used MmCpn since it can function at ordinary temperatures. In contrast to our expectations, MmCpn exhibited only a slight motion in the DXT experiment. In our previous DET study using gold nanocrystals, we immobilized the crystals on a carbon film to reduce the attenuation of the electron beam in the buffer. However, we abandoned such configuration because it increases the electron beam-induced protein damage. Therefore, we immobilized MmCpn on the bottom of the EC to expose gold nanorods close to the carbon thin film. The sample setup worked well, and the motions of gold nanorods were clearly traced. Compared with the results for DXT, the mobility in DET was significantly higher. This finding seems to be due to the difference in the method for immobilization. For DXT, chaperonins were directly immobilized on the solid surface. Eight cysteine residues were located on each side of a chaperonin, and then chaperonin was rigidly immobilized, which might repress the motion of chaperonin. In the case of TKS1-Cpn, the mobility was sufficiently high to be observed in such immobilization condition as the reaction temperature was high. On the contrary, in DET, MmCpn was immobilized on the TAC, which was bound to the solid support. Therefore, MmCpn could move relatively freely compared with the motion in DXT. Although we have already shown the advantages of DXT for tracing the motions of proteins [11,13], DXT has not widely used because of the limited availability of X-ray sources and the need for gold nanocrystals. This study has shown that DET can resolve the above problems of DXT. DET should be a state-of-the-art technology for analyzing protein dynamics.

## 4. Materials and Methods

### 4.1. Expression and Purification of MmCpnK256C and MmCpnP255C

The genes for MmCpnK256C and MmCpnP255C were made by site-directed mutagenesis of the gene of the Cys-less mutant of MmCpn. The MmCpn variants were overproduced in *E. coli* strain Rosetta (DE3) pLysS using a pET21 vector (Merck Millipore; Billerica, MA, USA). The cells were harvested by centrifugation, suspended in buffer A (50 mM Tris-HCl (pH 7.5), 5 mM MgCl_2_, and 1 mM dithiothreitol) and disrupted by sonication. The lysate was centrifuged at 15,000× *g* for 30 min to pellet the cell debris. The supernatant was loaded on a TOYOPEARL SuperQ-650 column (TOYOBO; Osaka, Japan) equilibrated with buffer A. Bound proteins were eluted by a NaCl gradient ranging from 0 to 500 mM. Fractions containing MmCpn variants were pooled and loaded on an AF-Heparin HC-650M (TOYOBO) equilibrated in buffer A with 0.28 M NaCl. Bound proteins were eluted by a NaCl gradient ranging from 0.28 to 1 M. Fractions containing MmCpn variants were pooled and concentrated using an Amicon Ultra-15 10K concentrator (Merck Millipore). Then, the oligomers of MmCpn variants were purified by size exclusion chromatography using a HiLoad 26/60 Superdex200 pg (GE Healthcare, Buckinghamshire, UK) with Buffer B (buffer A + 150 Mm NaCl). Fractions containing the oligomers of MmCpn variants were collected.

### 4.2. Fluorescence Intensity Assay

Cys256 of MmCpnK256C was labeled with fluorescein 5-maleimide (Invitrogen; Carlsbad, CA, USA). The fluorescence spectra were measured at 29 °C with a FP-6500 Spectrofluorometer (JASCO; Tokyo, Japan). MmCpnK256C complexes (50 nM) in TKM buffer were pre-incubated with or without ATP (1 mM) at 29 °C. The excitation wavelength was set at 493 nm, and the fluorescence was recorded from 500 to 650 nm.

### 4.3. DXT Measurement

A gold substrate was prepared by vapor deposition coating of a 50-μm thick polyimide film (Kapton®, Du Pont-Toray; Tokyo, Japan) with chromium (10 nm) and gold (25 nm). An aliquot of MmCpnP255C solution (0.2 mg/mL) in MOPS buffer (50 mM MOPS, 100 mM KCl, and 5 mM MgCl_2_, pH 7.0) was applied to the gold substrate for 2 h at 4 °C. Thus, prepared MmCpn modified substrate was reacted with the gold nanocrystal solution for 1–2 h at 4 °C. After removal of the excess gold nanocrystals by the rinse with MOPS buffer, the gold nanocrystal-modified MmCpn substrate was stored in the MOPS buffer until use. An experimental chamber was constructed of the sample substrate film with a spacer of a 50-μm thick polyimide film. The chamber was filled with MOPS buffer with or without 1 mM ATP for DXT measurement.

X-rays of 14.0–16.5 keV (Undulator ID gap = 31.0 mm) from beam line BL40XU (SPring-8; Sayo, Japan) were used to record the Laue diffraction spots from the gold nanocrystals on chaperonins. The sizes of the X-ray beam on the sample were 50 µm (vertical) and 50 µm (horizontal). The time-resolved diffraction images were monitored using an X-ray image intensifier (V5445P, Hamamatsu Photonics, Hamamatsu, Japan) and a CMOS camera (C11440-10C, Hamamatsu Photonics) with a 40 ms/f frame rate for 3.6 s. The distance between the sample substrate and the detector was approximately 100 mm and calibrated by diffraction from a gold film. The sample temperature during DXT was controlled to approximately 30 °C by hot air blowers (TRIAC PID; Leister, Kaegiswil, Switzerland). Gold nanocrystals were prepared as described previously [11]. The average diameter of the gold nanocrystals was estimated to be 40 nm and confirmed by AFM images. Custom software written for IGOR Pro (Wavemetrics; Lake Oswego, OR, USA) was used to analyze the diffracted spot tracks and trajectories.

### 4.4. Sample Preparations for DET

A film of triacetyl cellulose (TAC) was used to support the MmCpnP255C. A 1% (*w*/*v*) TAC (Okenshoji Ltd., Tokyo, Japan) solution was prepared by dissolving TAC in a mixed solvent of 90% (*v*/*v*) 1,2-dichloroethane and 10% (*v*/*v*) methanol. A glass slide was soaked in the TAC solution and extracted at 2.5 mm/s. The TAC film was removed from the glass slide on the surface of the water and placed on the 50-µm thick polyimide film (Kapton^®^, Du Pont-Toray, Tokyo, Japan) ring (outer diameter 1.5 mm, inner diameter 1 mm). The polyamide ring with the TAC film was removed from the water and dried. Then, thin gold islands (several Å) were prepared on the TAC film by vapor deposition. Two microliters of MmCpnP255C in TKM buffer (5 µg/mL) was dropped onto the TAC film and incubated for 2 h at 4 °C. After two washes with TKM buffer, 2 µL of the gold nanorod (Axial Diameter 25 nm, Longitudinal Size 34 nm, Strem Chemicals Inc.; Newburyport, MA, USA) suspension in TKM buffer was dropped. After further incubation for 2 h at 4 °C, excess gold nanorod was removed by washing with TKM buffer twice. Finally, TKM buffer with or without 1 mM ATP was dropped on the thus prepared TAC support with samples, and then the support was placed into the EC. The EC was assembled from a 150-m diameter single-hole grid (Agar Scientific; Essex, UK) and an O-ring (inner diameter 2.0 mm, thickness 0.45 mm) at the top of the EC holder.

### 4.5. DET Measurement 

A JSM-7001F SEM system (JEOL, Tokyo, Japan), equipped with a Schottky-type field emission electron gun was used in the DET experiment. To obtain the EBSP signal, we used an electron beam of 30 keV accelerating voltage, an 87 pA beam current, and a 17 mm working distance at a 70-degree angle relative to the EC sample stage. We detected EBSDs by the EBSD detector on the JSM-7001F system, which was custom designed by TSL Solutions (DVC1412-FW-T1-EX, TSL Solutions; Sagamihara, Japan). The detector included an image intensifier (V8070U-74, Hamamatsu Photonics; Hamamatsu, Japan). Each spot was irradiated with the electron beam for 1.5 s. The EBSP was obtained on the phosphor screen of the detector, intensified, and recorded with a CCD camera using a 60 ms shutter speed. The Euler angles, which indicate the rotational relationship between the sample stage coordinate system and the crystal lattice coordinate system of the gold nanorod, were obtained from the EBSP using OIM Analysis (EDAX Inc.; Mahwah, NJ, USA). From the Euler angles of adjacent EBSP frames, the single-axis rotation angle ω and the angles *α*, *β*, and *γ* of the principal lattice vectors *a*, *b*, and *c*, were calculated as described previously [15].

## 5. Conclusions

In this study, we first demonstrated the analysis of the protein dynamics by DET. We could observe ATP-dependent rotation motion of the group II chaperonin from the mesophilic archaeon *M. maripaludis*, by immobilizing the chaperonin on the triacetyl cellulose support. Therefore, we could observe the EBSP from gold nanorods with reduced irradiation damage of the protein. The immobilization method is also advantageous compared to the method used for DXT since it gives relatively high mobility. Although much remains to do for developing DET, DET should be a state-of-the-art technology for analyzing protein dynamics.

## Figures and Tables

**Table 1:** Angular/rotational diffusion constant calculated from MSD.

Condition	*D* (rad^2^/s)
*θ* without ATP	5.7 × 10^−6^
*θ* with ATP	6.9 × 10^−6^
*χ* without ATP	6.4 × 10^−6^
9.0 × 10^−6^	*χ* with ATP

The values were obtained from the slope of the MSD versus time plot shown in Figure 2. The lines in Figure 2B were fitted with least-squares fitting to the following equation, *MSD* = 4*Dt* + *a*, where MSD is the mean square angular displacement, *D* is the angular/rotational diffusion constant, *t* is time interval, and *a* is intercept.

## Abbreviations

DXT	Diffracted X-ray tracking
DET	Diffracted electron tracking
MmCpn	Group II chaperonin from *Methanococcus maripaludis*
TKS1-Cpn	Group II chaperonin from a hyperthermophilic archaeon, *Thermococcus* sp. strain KS1
EC	Environmental cell
SEM	Scanning Electron Microscope
TAC	Triacetyl cellulose
